# The dynamics of plasma biomarkers across the Alzheimer’s continuum

**DOI:** 10.1186/s13195-023-01174-0

**Published:** 2023-02-08

**Authors:** Yu Guo, Xue-Ning Shen, Hui-Fu Wang, Shi-Dong Chen, Ya-Ru Zhang, Shu-Fen Chen, Mei Cui, Wei Cheng, Qiang Dong, Tao Ma, Jin-Tai Yu

**Affiliations:** 1 Department of Neurology and Institute of Neurology, Huashan Hospital, State Key Laboratory of Medical Neurobiology and MOE Frontiers Center for Brain Science, Shanghai Medical College, Fudan University, National Center for Neurological Disorders, Shanghai, China; 2 Institute of Science and Technology for Brain-Inspired Intelligence, Fudan University, Shanghai, China; 3 Key Laboratory of Computational Neuroscience and Brain-Inspired Intelligence, Fudan University, Ministry of Education, Shanghai, China; 4 Fudan ISTBI—ZJNU Algorithm Centre for Brain-inspired Intelligence, Zhejiang Normal University, Jinhua, China; 5 Department of Neurology, Wuxi Second People Hospital, Jiangnan University Medical Center, Wuxi, China

**Keywords:** Alzheimer’s continuum, Plasma, Biomarker, Trajectory, Glial fibrillary acidic protein

## Abstract

**Background:**

Failures in drug trials strengthen the necessity to further determine the neuropathological events during the development of Alzheimer’s disease (AD). We sought to investigate the dynamic changes and performance of plasma biomarkers across the entire Alzheimer’s continuum in the Chinese population.

**Methods:**

Plasma amyloid-β (Αβ)42, Aβ40, Aβ42/Aβ40, phosphorylated tau (p-tau)181, neurofilament light (NfL), and glial fibrillary acidic protein (GFAP) were measured utilizing the ultrasensitive single-molecule array technology across the AD continuum (*n*=206), wherein Aβ status was defined by the values of cerebrospinal fluid (CSF) Aβ42 or Aβ positron emission tomography (PET). Their trajectories were compared with those of putative CSF biomarkers.

**Results:**

Plasma GFAP and p-tau181 increased only in Aβ-positive individuals throughout aging, whereas NfL increased with aging regardless of Aβ status. Among the plasma biomarkers studied, GFAP was the one that changed first. It had a prominent elevation early in the cognitively unimpaired (CU) A+T− phase (CU A+T− phase: 97.10±41.29 pg/ml; CU A−T− phase: 49.18±14.39 pg/ml; *p*<0.001). From preclinical to symptomatic stages of AD, plasma GFAP started to rise sharply as soon as CSF Aβ became abnormal and continued to increase until reaching its highest level during the AD dementia phase. The greatest slope of change was seen in plasma GFAP. This is followed by CSF p-tau181 and total-tau, and, to a lesser extent, then plasma p-tau181. In contrast, the changes in plasma NfL, Aβ42/Aβ40, Aβ42, and Aβ40 were less pronounced. Of note, these plasma biomarkers exhibited smaller dynamic ranges than their CSF counterparts, except for GFAP which was the opposite. Plasma GFAP and p-tau181 were tightly associated with AD pathologies and amyloid tracer uptake in widespread brain areas. Plasma GFAP could accurately identify CSF Aβ42 (area under the curve (AUC)=0.911) and Aβ PET (AUC=0.971) positivity. Plasma p-tau181 also performed well in discriminating Aβ PET status (AUC=0.916), whereas the discriminative accuracy was relatively low for other plasma biomarkers.

**Conclusions:**

This study is the first to delineate the trajectories of plasma biomarkers throughout the Alzheimer’s continuum in the Chinese population, providing important implications for future trials targeting plasma GFAP to facilitate AD prevention and treatment.

**Supplementary Information:**

The online version contains supplementary material available at 10.1186/s13195-023-01174-0.

## Background

As a leading cause of dementia, Alzheimer’s disease (AD) is quickly becoming one of the most expensive, lethal, and burdening diseases of the 21st century [1]. China has the largest population of patients with AD, with an estimated 9.83 million individuals aged 60 years or older [2]. Unfortunately, numerous drug candidates for AD have not been successful, and no approved therapies exist at present. This grim situation reinforces the necessity of accurately detecting the neuropathological events (especially the earliest events) in AD, which is a basis for the selection of individuals eligible for treatments [1].

The pathophysiological changes of AD, notably amyloid β (Aβ)-containing senile plaques and tau-containing neurofibrillary tangles, occur years before symptoms arise [3–5]. Current cerebrospinal fluid (CSF) and positron emission tomography (PET) assays enable the robust assessment of classic pathophysiological hallmarks of AD [1, 6]. However, the roll-out of these technologies into clinical practice has been hampered by their high costs, invasiveness, and unavailability in routine settings [7]. Blood tests, which circumvent the aforementioned drawbacks of CSF and PET biomarkers, are gaining increasing attention due to their great potential for real-world implementation [8]. Indeed, several research groups have investigated blood measures of Aβ pathology (Aβ42/Aβ40, Aβ42, and Aβ40), tau deposition (phosphorylated tau181 (p-tau181)), neurodegeneration (neurofilament light (NfL)), and glial activation (glial fibrillary acid protein (GFAP)), and discovered important clues for AD diagnosing, screening and monitoring [1, 9–18].

Nevertheless, the patterns of dynamic changes of various blood biomarkers across the whole Alzheimer’s continuum remain to be explored. On the one hand, recruiting individuals from the preclinical to symptomatic phases of AD is particularly challenging. Several previous studies, restricted by unavailable CSF or PET data, could only focus on the clinical spectrum of AD [11, 19, 20] or simply classify the Alzheimer’s continuum into A− and A+ stages [10, 12]. On the other hand, many studies included a single or very low number of markers but ignored the head-to-head comparison with other biomarkers [11, 21], hence the relative orderings of biomarker changes couldn’t be derived. Recent studies overcame both of the above shortcomings, but they examined only the preclinical stage of AD [22] or preclinical and prodromal stages [23] and lacked an understanding of the whole AD continuum. In addition, these former studies are primarily from western countries, how early the blood biomarkers begin to change, their characteristics of alterations, and whether these biomarkers reflect AD pathologies within the Chinese population is unclear. Because of recent reports on potential racial differences in AD biomarkers [24–26], our study of Chinese cohorts may provide novel insights.

Herein, we aimed to perform a comprehensive comparison of changes in plasma Aβ42/Aβ40, Aβ42, Aβ40, p-tau181, NfL, and GFAP in the context of established CSF biomarkers from the asymptomatic preclinical stage to symptomatic phases of AD. Moreover, we investigated the abilities of these plasma biomarkers to indicate AD pathophysiology. By addressing the above issues, we expected to provide a reference for understanding the main pathophysiological changes and, concomitantly, for better informing the design of clinical trials of AD.

## Methods

### Study population

Data used in the present study were obtained from the Chinese Alzheimer’s Biomarker and LifestylE (CABLE) cohort (recruitment site: Qingdao Municipal Hospital, Qingdao, China; detailed information was reported previously [27, 28]) and the Department of Neurology, Huashan Hospital, Fudan University (Shanghai, China). We considered all consecutive participants with both available plasma and CSF or PET examinations who had a first diagnostic visit between November 2017 and May 2021. All participants were Chinese Han adults aged between 40 and 90 years and underwent comprehensive clinical and neuropsychological evaluations. The exclusion criteria include (a) central nervous system infections, head trauma, other neurodegenerative disorders (e.g., Parkinson’s disease), or other major neurological disorders; (b) major psychological diseases; (c) severe systemic diseases (e.g., cancer); and (d) family history of genetic diseases. The study was conducted following the Helsinki Declaration and approved by the institutional medical ethical committees. All participants or their authorized representatives provided written consent.

Cognitively unimpaired (CU), mild cognitive impairment (MCI), and AD participants were included and further categorized according to the NIA-AA research framework [6]. In the CABLE cohort, there were 93 CU and 54 MCI individuals receiving lumbar punctures, and the results of CSF Aβ42 and p-tau181 were used to define Aβ and tau status respectively. In Huashan Hospital, another 30 MCI patients underwent PET examinations, and Aβ status was defined by the results of Aβ PET. Patients with AD dementia (*n*=93) from Huashan Hospital were diagnosed with confirmed evidence of both abnormal Aβ and tau PET burden. Accordingly, CU individuals were classified into A−T−, A+T−, A+T+, and A−T+ groups. Participants within CU A+T− and CU A+T+ groups together belonged to the preclinical AD stage [29]. MCI individuals with Aβ positivity were classified into the MCI due to AD stage [30, 31]. Individuals within the preclinical AD stage and the MCI due to AD stage, together with AD dementia patients, fell into the Alzheimer’s continuum [6]. In addition, patients with non-AD dementia (frontotemporal dementia (FTD) (*n*=31) and vascular dementia (VaD) (*n*=37)) were included from Huashan Hospital for supplementary analysis.

### Sample collection and biomarker measurements

Using a uniform preanalytic protocol, all venous fasting blood samples were collected by board-certified laboratory technicians blinded to clinical information. After resting at room temperature for 30 min, these samples were centrifuged at 1800 rpm at 4 °C for 15 min. The supernatant was then divided into 200 μL aliquots and immediately frozen at −80°C until assay. All blood samples were transferred to Huashan Hospital, and then uniformly quantified by ultrasensitive single-molecule array (Simoa) technology (Quanterix, Billerica, MA, USA) on the automated Simoa HD-X platform. Plasma Aβ42, Aβ40, NfL, and GFAP levels were measured using the Simoa® Neurology 4-Plex E assay (catalog number: 103670), and p-tau181 levels were measured with the Simoa^TM^ pTau-181 Advantage V2 assay (Catalog Number: 103714). The within- and inter-batch coefficients of variations for all assays were <10% and <20%, respectively. The variations of all samples were below this quality control standard. The analytical lowest limit of quantification was 0.378 pg/mL for Aβ42, 1.020 pg/mL for Aβ40, 0.400 pg/mL for NfL, 2.890 pg/mL for GFAP, and 0.085 pg/mL for p-tau181. All specimens tested exceeded these thresholds. Besides, quality control analysis showed that the plasma levels were not related to sampling collection sites.

CSF Aβ42, Aβ40, p-tau181, and total-tau (t-tau) were quantified by the custom ELISA assays as previously described [27, 28]. The optimal cut-off points for CSF Aβ42 (194.50 pg/mL) and p-tau181 (57.50 pg/mL) were determined by the Youden index. More information was shown in Supplementary Methods. For comparison purposes, CSF GFAP and NfL levels were also quantified using the Simoa® Neurology 4-Plex E assay (catalog number: 103670).

### Image acquisition and processing

The maximum interval between plasma tests and imaging scans was 10 days. Participants underwent amyloid (^18^F-AV45) and tau (^18^F-APN-1607) PET scans after a cranial T1-weighted structural magnetic resonance imaging (MRI) scan on a 3.0 T MRI scanner (Discovery 750, GE Healthcare, Milwaukee, WI, USA) in the PET Center of Huashan Hospital, Fudan University. PET tracer uptake was measured at 50 min and 90 min after intravenous injection of ^18^F-AV45 and ^18^F-APN-1607, respectively. The acquisition time was 20 min. PET images were then motion-corrected, time-averaged, and thoroughly co-registered with their corresponding T1-MRI images using Statistical Parametric Mapping version 12. Amyloid and tau positivity was determined by visual inspection and semiquantitative assessments. Standardized uptake values were extracted using the cerebellum gray matter as a reference region. Partial volume correction was applied under the Geometric Transfer Matrix model.

### Statistical analyses

Data were initially screened for outliers and those that fell outside five standard deviations (SDs) were removed. Baseline characteristics across different groups were compared by the Chi-square test or Fisher's exact test for categorical variables and the Kruskal-Wallis test for continuous variables. Plasma and CSF biomarkers were tested for normality using the Kolmogorov-Smirnov test and visual inspection of histograms. All biomarkers had skewed distributions and were thus log_10_-transformed.

To exhibit the distributions of plasma levels, the plots delineated raw values, although group comparisons were done using the one-way analysis of covariance adjusted for age and sex. The Spearman rank test was utilized for correlations between plasma biomarkers. Associations of each plasma biomarker with age, sex, education, and *APOE* ε4 status were analyzed using multiple linear regressions. Additionally, we performed these analyses stratifying by Aβ status and including in the regression model an “age×Aβ42” interaction term.

To test whether plasma biomarkers were associated with AD pathologies, multiple linear regression analyses were performed. Receiver operating curve (ROC) analyses were applied to estimate the discriminative ability of age- and sex-corrected levels of the plasma biomarkers. The R package “pROC” was used for the visualization of the curves. The area under the curve (AUC), sensitivity, and specificity were reported for each biomarker.

As performed elsewhere [12, 32], we corrected biomarker values by age and sex and converted them to z-scores anchored on the normative data of the CU A−T− group (reference group). The associations between each biomarker and disease progression or the proxies of disease progression (i.e., CSF Aβ42/Aβ40 and p-tau) were modeled using the robust local weighted regression method. We then computed the linear regression slopes for biomarkers in each of the negative or positive groups and tested whether the slopes were statistically significantly different.

All statistical analyses were carried out using R version 4.1.2 (http://www.r-project.org/). A two-tailed p-value <0.05 was deemed statistically significant. The false discovery rate (FDR) correction method was applied for multiple corrections except where specifically noted.

## Results

### Participants’ characteristics

The demographic, clinical, and fluid biomarker features of included participants were summarized in Table 1 and Table S1. The diagnostic groups differed in years of age, the prevalence of *APOE* ε4 positivity, and cognitive scores, but not in sex distribution, education levels, or family history. Since we aimed to study the Alzheimer’s continuum, those who fell into CU A−T+ and MCI− groups (non-AD pathologic changes) were removed from all analyses, and they were only shown for descriptive purposes.

**Table 1:** Demographic, clinical characteristics, and biomarker patterns per diagnostic group

Characteristic	CU A−T− *N* = 43	CU A+T− *N* = 17	CU A+T+ *N* = 17	CU A−T+ *N* = 16	MCI− *N* = 48	MCI+ *N* = 36	AD dementia *N* = 93	Non-AD dementia *N* = 68	*P*-value
Age, years	57.02 (6.76)	61.65 (9.56)	68.06 (10.87)	58.88 (7.21)	61.69 (10.71)	66.72 (9.82)	59.48 (8.28)	62.41 (8.70)	<0.001
Male	18 (41.86%)	10 (58.82%)	7 (41.18%)	6 (37.50%)	25 (52.08%)	15 (41.67%)	38 (40.86%)	40 (58.82%)	0.303
Education, years	10.49 (4.22)	9.00 (3.00)	7.35 (4.86)	11.06 (3.43)	8.91 (3.72)	9.61 (3.11)	8.52 (4.26)	8.52 (4.00)	0.057
*APOE* ε4 carrier	3 (7.14%)	3 (18.75%)	3 (17.65%)	2 (12.50%)	12 (29.27%)	8 (24.24%)	33 (37.50%)	15 (28.85%)	<0.001
Family history	1 (2.33%)	0 (0.00%)	0 (0.00%)	0 (0.00%)	5 (11.11%)	3 (9.09%)	4 (4.30%)	4 (6.35%)	0.401
MMSE score	28.70 (1.63)	28.29 (1.53)	26.35 (2.78)	28.38 (1.45)	25.31 (4.39)	25.40 (3.47)	13.76 (6.20)	16.52 (7.71)	<0.001
MoCA score	25.33 (3.01)	25.40 (2.50)	22.50 (4.66)	26.00 (2.39)	18.04 (5.02)	18.29 (4.21)	8.16 (5.77)	10.44 (6.80)	<0.001
Aβ-PET SUVRs	–	–	–	–	0.98 (0.09)	1.06 (0.10)	1.54 (0.30)	–	<0.001
**Plasma biomarker**									
GFAP, pg/ml	49.18 (14.39)	97.10 (41.29)	106.64 (40.73)	49.71 (13.93)	68.10 (29.53)	123.18 (54.82)	262.66 (92.77)	132.18 (68.94)	<0.001
P-tau181, pg/ml	1.92 (0.78)	1.91 (0.58)	2.92 (1.39)	1.99 (0.67)	1.99 (0.89)	2.88 (1.70)	4.60 (1.82)	2.08 (1.16)	<0.001
Aβ42/Aβ40	0.07 (0.03)	0.06 (0.01)	0.05 (0.01)	0.06 (0.02)	0.06 (0.01)	0.05 (0.01)	0.05 (0.01)	0.06 (0.01)	<0.001
Aβ42, pg/ml	4.85 (1.98)	5.17 (1.31)	4.41 (0.99)	4.78 (1.22)	4.91 (1.39)	4.92 (2.53)	4.37 (1.12)	5.45 (1.76)	0.003
Aβ40, pg/ml	74.94 (25.62)	88.01 (21.57)	89.20 (23.08)	83.04 (28.28)	84.55 (26.24)	96.07 (54.98)	94.83 (24.18)	94.08 (32.42)	0.010
NfL, pg/ml	11.99 (7.87)	14.12 (8.69)	19.88 (11.39)	13.03 (7.21)	17.27 (11.18)	36.05 (76.90)	28.76 (12.34)	66.28 (74.09)	<0.001
**CSF biomarker**									
Aβ42, pg/ml	327.31 (81.35)	153.56 (44.18)	150.19 (37.41)	371.14 (118.78)	391.93 (178.47)	165.10 (93.61)	–	–	<0.001
Aβ40, pg/ml	5553.03 (2168.36)	4561.35 (1742.51)	7808.73 (2596.85)	8116.11 (2409.33)	7420.14 (2586.36)	8128.43 (7620.42)	–	–	<0.001
Aβ42/Aβ40	0.07 (0.03)	0.04 (0.02)	0.02 (0.01)	0.05 (0.02)	0.06 (0.03)	0.03 (0.02)	–	–	<0.001
P-tau181, pg/ml	38.45 (7.13)	32.70 (8.69)	69.42 (18.04)	58.16 (8.40)	45.94 (13.50)	58.79 (25.74)	–	–	<0.001
Total-tau, pg/ml	155.34 (41.24)	153.56 (53.91)	345.96 (160.69)	230.80 (73.82)	239.77 (146.34)	328.01 (219.70)	–	–	<0.001

Continuous data are described as mean (standard deviations (SDs)), and categorical variables are presented as numbers (percentages). Non-AD dementia refers to frontotemporal dementia and vascular dementia. Patients with AD or non-AD dementia did not have available CSF AD core biomarker data. Only MCI−, MCI+, and AD dementia patients had available Aβ-PET data. Here, we showed average Aβ-PET SUVRs in the middle temporal lobe

*Abbreviations*: *CU* cognitively unimpaired, *MCI* mild cognitive impairment, *AD* Alzheimer’s disease, *APOE apolipoprotein E*, *MMSE* Mini-Mental State Examination, *MoCA* Montreal Cognitive Assessment, *Αβ* amyloid-β, *PET* positron emission tomography, *SUVRs* standard uptake value ratios, *GFAP* glial fibrillary acidic protein, *p-tau* phosphorylated tau, *NfL* neurofilament light, *CSF*, cerebrospinal fluid

### Group comparisons of plasma levels

**Fig. 1:**
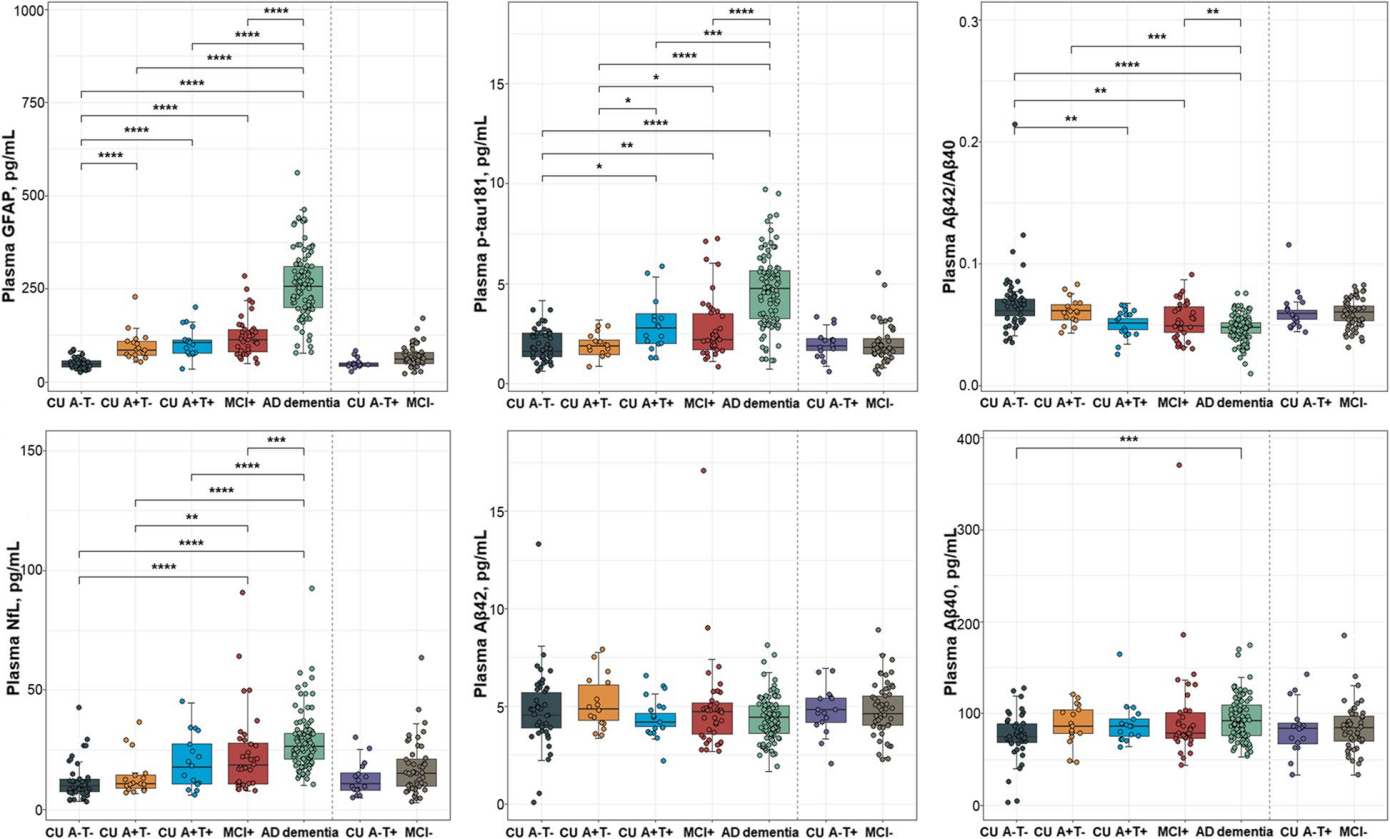
Group comparisons of plasma biomarkers. Plasma levels of GFAP, p-tau181, Αβ42/Αβ40, NfL, Αβ42, and Αβ40 between groups were compared with one-way analysis of covariance controlling for age and sex, followed by FDR corrected pair-wise post hoc comparisons. Significance: ****p<0.0001, ***p<0.001, **p<0.01, *p<0.05, −: *p*≥0.05. Abbreviations: GFAP, glial fibrillary acidic protein; p-tau, phosphorylated tau; Αβ, amyloid-β; NfL, neurofilament light; FDR, false discovery rate; MCI, mild cognitive impairment; AD, Alzheimer’s disease

The tested plasma markers showed different dynamic trends throughout the Alzheimer’s continuum (Fig. 1). Plasma GFAP levels were markedly elevated in the CU A+T− group relative to the CU A−T− group, whereas other plasma levels were comparable between the two groups. Compared with the CU A−T− group, the CU A+T+ group had significantly increased GFAP and p-tau181 levels as well as significantly reduced Aβ42/Aβ40 levels. As expected, similar trends were observed when comparing the MCI+ or AD dementia group versus the CU A−T− group. Compared with the CU A+T− group, only p-tau181 levels were higher in the CU A+T+ and MCI+ groups. In contrast, plasma NfL was not significantly elevated until the MCI+ stage, where NfL levels were higher than those in the CU A−T− or CU A+T− groups. Additionally, the AD dementia group had increased GFAP, p-tau181, and NfL levels relative to the CU A+T−, CU A+T+, and MCI+ groups, as well as decreased Aβ42/Aβ40 levels relative to the CU A+T− and MCI+ groups. Across the entire Alzheimer’s continuum, Aβ42 and Aβ40 levels were essentially unchanged, except that the AD dementia group had increased Aβ40 levels than the CU A−T− group. Comparisons with FTD and VaD groups were presented in Fig. S1. Correlations between plasma biomarkers were shown in Fig. S2.

### Plasma biomarkers and demographic factors

**Fig. 2:**
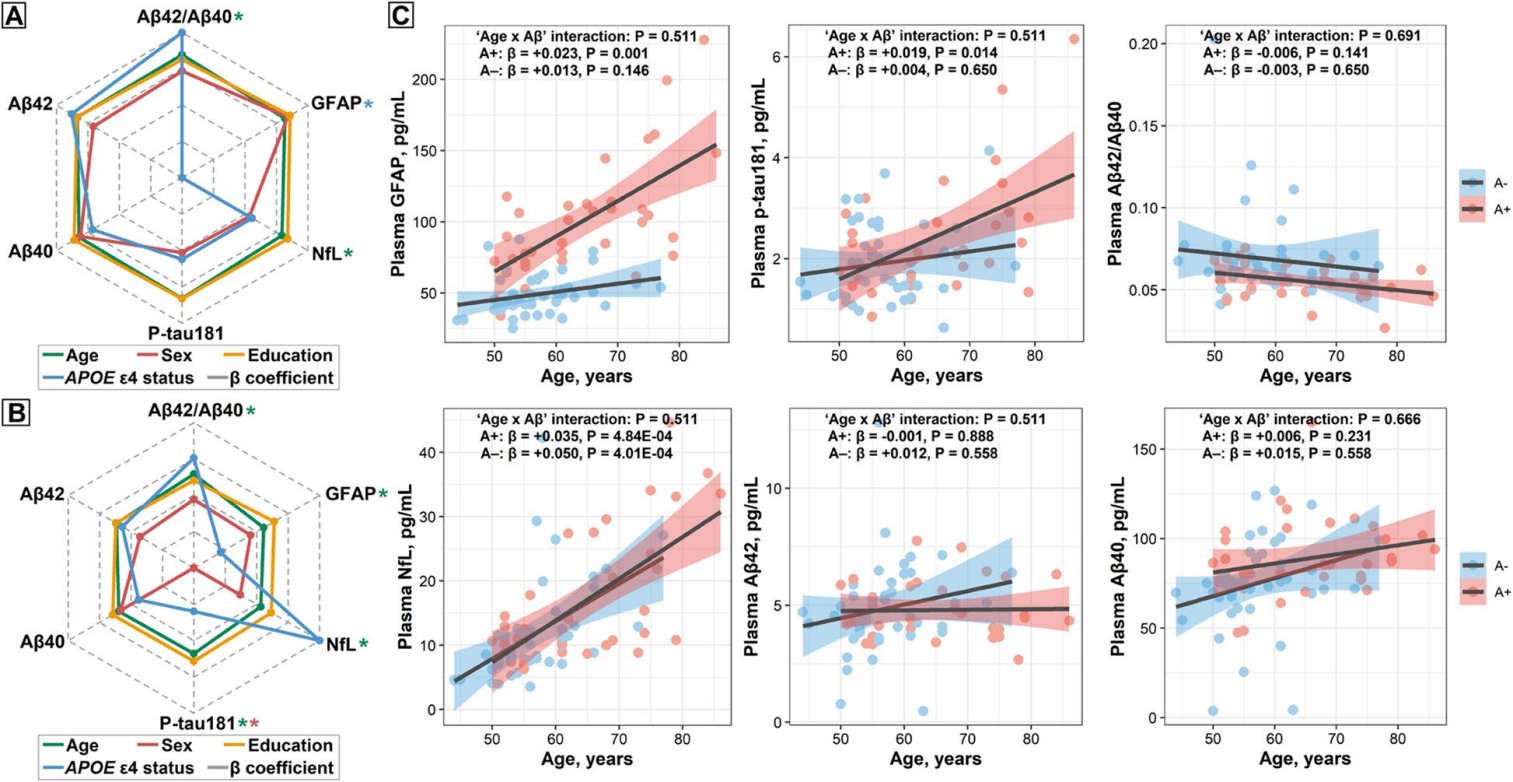
Correlations of plasma markers with demographic features. Demographic factors (age, sex, education, and *APOE* ε4 status) correlated to plasma measures (log-converted) were analyzed using multiple linear regressions. When one of the demographic factors was analyzed as a predictor variable, the other three factors were included in the model as covariates. The dotted gray lines from the center to the periphery represented the derived correlation coefficient (β) ranging from −0.080 to 0.405 in the CU A−T− and the whole Alzheimer’s continuum (CU A+T−, CU A+T+, MCI+, and AD dementia) groups (**A**) and from -0.161 to 0.271 in the CU A−T− and preclinical AD (CU A+T−, CU A+T+) groups (**B**). The * indicated the FDR-corrected *p*<0.05. **C** Scatter plots showing the associations of each plasma biomarker with age in the CU A−T− and preclinical AD groups. The standardized regression coefficients (β) and the *P*-values were computed using multiple linear regressions adjusting for sex, education, and *APOE* ε4 status. Additionally, we calculated the “age×Aβ status” interaction term. All P-values are corrected for multiple comparisons using the FDR approach. Abbreviations: *APOE*, *apolipoprotein E*; MCI, mild cognitive impairment; AD, Alzheimer’s disease; FDR, false discovery rate; Αβ, amyloid-β; GFAP, glial fibrillary acidic protein; NfL, neurofilament light; p-tau, phosphorylated tau

Among participants with normal AD biomarkers (CU A−T−) and those within the whole Alzheimer’s continuum (CU A+T*, MCI+, and AD dementia), plasma Aβ42/Aβ40 and NfL were significantly associated with age, while sex and education could not affect any of the plasma biomarkers tested (Fig. 2A). Plasma GFAP was associated with *APOE* ε4 status, but this association diminished after further adjusting for CSF Aβ42 levels, which indicated that the observed association might be driven by Aβ pathology.

For participants within CU A−T− and preclinical AD (CU A+T*) groups, plasma Aβ42/Aβ40, p-tau181, NfL, and GFAP were significantly associated with age (Fig. 2B). When stratifying by Aβ status, we observed that the alterations of plasma GFAP and p-tau181 as a function of age only occurred in the A+ group but not in the A− one, albeit the “age×Aβ” interaction term was non-significant (Fig. 2C). Besides, the positive effect of age on NfL levels was significant in both A+ and A− groups, but not for the rest of the plasma biomarkers. Plasma p-tau181 levels were higher in men, and including the CSF Aβ42 as a covariable did not change the results, which indicated that this observed difference between sexes was not driven by Aβ pathology. In contrast, education and *APOE* ε4 status were unrelated to any plasma biomarkers we tested (Fig. 2B).

### Associations of plasma levels with AD pathologies and discriminative accuracy

**Fig. 3:**
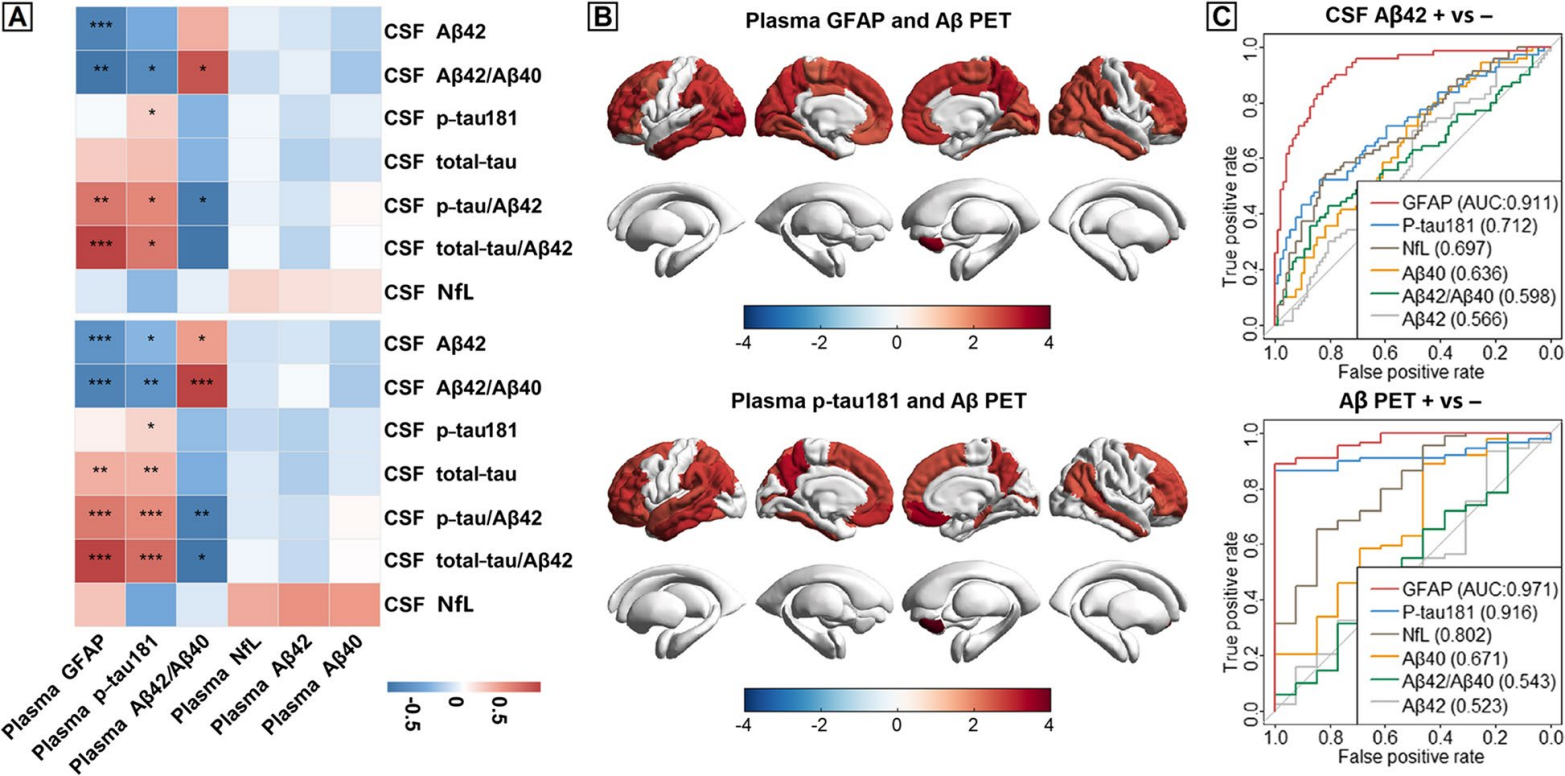
Associations of plasma biomarkers with AD pathologies and discrimination of Aβ status. Associations of plasma biomarkers with AD pathologies were tested using multiple linear regressions adjusted for age and sex. **A** Associations of plasma levels with CSF AD core biomarkers were tested in CU A−T− and preclinical AD (CU A+T−, CU A+T+) groups (the upper part) and in CU A−T− and prodromal AD (CU A+T−, CU A+T+, and MCI+) groups (the lower part). Colors represent correlation coefficients (β) in regression models and the color bar represents the range of β values obtained. Significance: ***p<0.001, **p<0.01, *p<0.05, −: *p*≥0.05 (FDR-corrected). **B** Relationships between plasma levels and cerebral amyloid burden as assessed on Aβ (^18^F-AV45) PET images. Aβ PET standard uptake value ratios (SUVRs) of different brain regions were extracted beforehand. Plasma GFAP and p-tau181 showed remarkably positive associations with Aβ PET SUVRs in many brain regions among MCI+ and AD dementia patients. Colors represent *t* values in regression models and the color bar represents the range of *t* values obtained. **C** Receiver operating curve analyses to discriminate Aβ-positive from Aβ-negative status. The upper and bottom plots showed the AUCs of different plasma biomarkers (plasma Aβ42/Aβ40, Aβ42, Aβ40, p-tau181, NfL, and GFAP) in discriminating Aβ status defined by CSF Aβ42 and Aβ PET, respectively. Abbreviations: AD, Alzheimer’s disease; Αβ, amyloid-β; CSF, cerebrospinal fluid; MCI, mild cognitive impairment; FDR, false discovery rate; GFAP, glial fibrillary acidic protein; p-tau, phosphorylated tau; PET, positron emission tomography; CU, cognitively unimpaired; NfL, neurofilament light; AUC, area under the curve

We evaluated the associations of plasma levels with AD pathologies as measured with CSF Aβ42, Aβ42/Aβ40, p-tau181, t-tau, p-tau/Aβ42, t-tau/Aβ42, NfL (Fig. 3A), and Aβ PET (Fig. 3B). Only three plasma biomarkers, comprising GFAP, p-tau181, and Aβ42/Aβ40, were significantly associated with CSF AD core biomarkers. Likewise, only GFAP and p-tau181 showed remarkably positive associations with amyloid tracer uptake in widespread brain areas (Table S2). Moreover, plasma GFAP could accurately differentiate CSF Aβ42 (AUC=0.911) and Aβ PET (AUC=0.971) status (Fig. 3C and Table S3). Plasma p-tau181 also performed well in discriminating Aβ PET status with an AUC of 0.916, whereas for other plasma biomarkers, the discriminative accuracy was relatively low. These ROC results were replicated when using CSF Aβ42/Aβ40 to define Aβ status (Table S3). Similar results were derived when contrasting CU A−T− with AD dementia individuals, and AD dementia with non-AD dementia patients (Fig. S3).

### Pathophysiological model of biomarker changes in the Alzheimer’s continuum

**Fig. 4:**
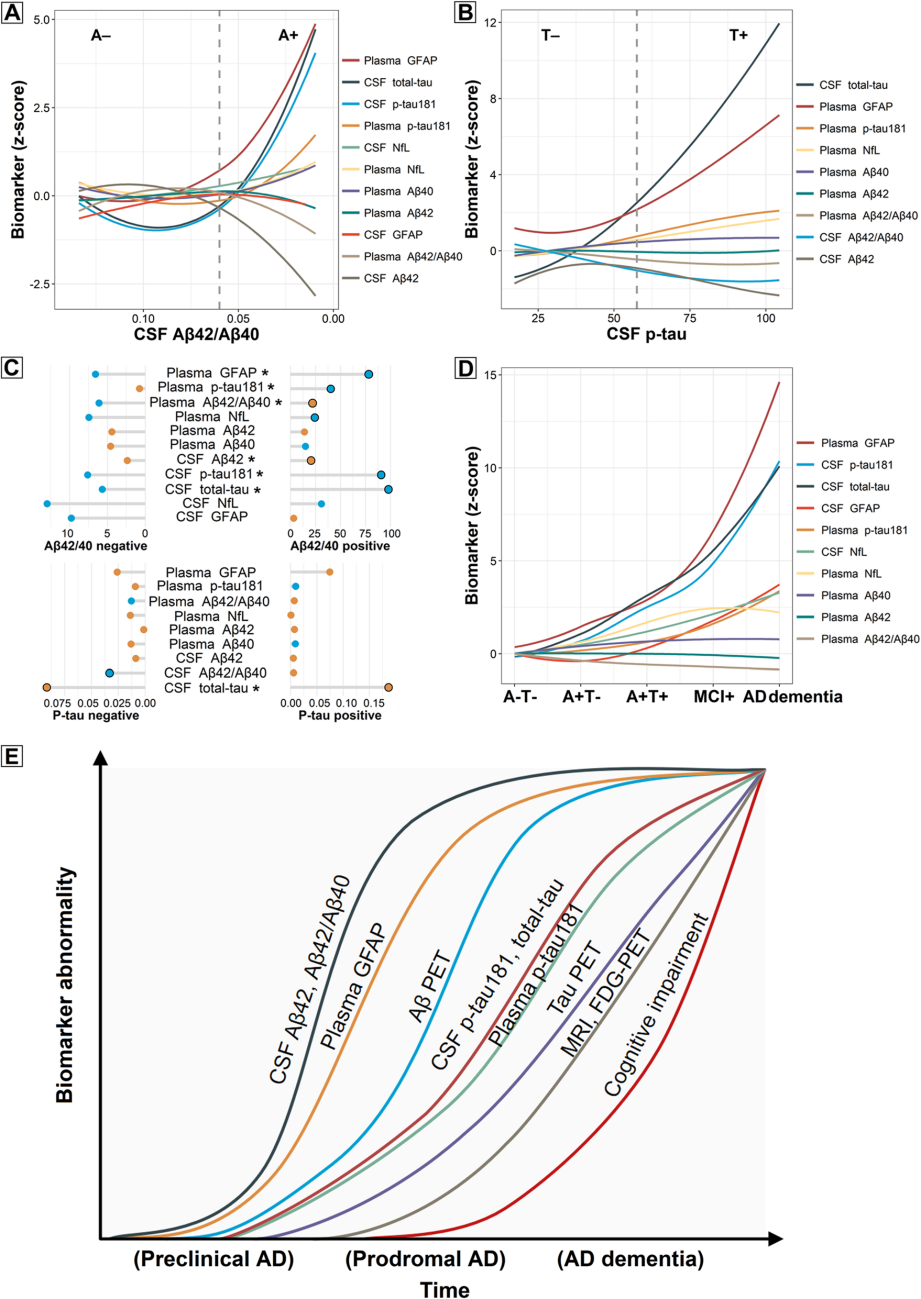
Trajectories of the biomarkers. Dynamic changes of the biomarkers as a function of CSF Aβ42/Aβ40 (**A**) or p-tau181 (**B**) in CU A−T− and preclinical AD stages. The graphs represent the z-score changes of plasma biomarkers using the CU A−T− group as a reference. For comparison purposes, the trajectories of CSF biomarkers were delineated. The solid curves depict biomarker trajectories using robust local weighted regressions. The dashed lines depict the cutoffs for CSF Aβ42/Aβ40 and p-tau. The horizontal axis direction of CSF Aβ42/Aβ40 (**A**) was inverted. **C** The relationship between each biomarker and CSF Aβ42/Aβ40 and p-tau181 in CU A−T− and preclinical AD individuals. For each biomarker, we computed the linear regression coefficients (β) and *p*-values of the *z*-scores in each of the negative or positive groups. The horizontal grey line represents the absolute value of β, and the color of the solid circle represents the positive (orange) or negative (blue) direction of β. The black circle outside the solid circle indicates the regression *p*-value<0.05. When the regression slopes between the negative and positive groups were different, a “*” was marked. **D** Dynamic changes of the biomarkers from CU A−T− to CU A+T*, MCI+, and AD dementia stages. The *z*-score changes of biomarkers using the CU A−T− group as a reference were delineated. **E** A model of the approximative orderings of AD-related biomarkers. Approximate stages of the Alzheimer’s continuum (preclinical, prodromal, and AD dementia) were shown on the x-axis. Considering personal reserves and vulnerability factors, we acknowledge that there may be substantial interindividual variations in the timing of different events. Abbreviations: CSF, cerebrospinal fluid; Αβ, amyloid-β; p-tau, phosphorylated tau; AD, Alzheimer’s disease; GFAP, glial fibrillary acidic protein; PET, positron emission tomography; NfL, neurofilament light; MCI, mild cognitive impairment; MRI, magnetic resonance imaging; FDG, fluorodeoxyglucose

To explore the earliest pathophysiological changes in the AD continuum, the trajectories of plasma biomarkers across preclinical AD were modeled using CSF Aβ42/Aβ40 (Fig. 4A) and p-tau181 (Fig. 4B) as proxies of disease progression. For comparison purposes, the ordinal sequences of CSF biomarkers were also mapped. Using CSF Aβ42/Aβ40 as a proxy of disease progression, we observed that plasma GFAP and p-tau181 began to prominently increase as soon as the CSF Aβ42/Aβ40 ratio became positive and continued to increase across the preclinical Alzheimer’s continuum, eventually reaching the highest *z*-scores (5 *z*-score for GFAP and 1.5 *z*-score for p-tau181) from their basal levels. These slopes in the preclinical stage were significantly different from the slopes of the CU A−T− stage. After Aβ positivity was reached, the greatest change for plasma biomarkers was seen for GFAP, followed by p-tau181, whereas the increase of NfL and Aβ40, as well as the decrease of Aβ42/Aβ40 and Aβ42, was considerably less pronounced (<1 *z*-score; as shown in the absolute slopes in Fig. 4A and consistent with the standardized regression coefficients (β) in Fig. 4C). In comparison, when the same z-score increase was achieved, the corresponding CSF Aβ42/Aβ40 values were highest for plasma GFAP, followed by CSF p-tau181 and t-tau, and then plasma p-tau181. Of note, the higher the corresponding CSF Aβ42/Aβ40 ratio, the earlier it is in the Alzheimer’s continuum.

Anchoring to CSF p-tau as a proxy of disease progression, we observed that plasma GFAP dramatically elevated before CSF p-tau positivity (Fig. 4B, C). Importantly, plasma GFAP levels already exceeded 1 *z*-score compared with the basal levels at the point when CSF p-tau became positive, indicating that the alterations of plasma GFAP preceded those of tau pathology. Plasma p-tau181 and NfL also increased as a function of CSF p-tau, eventually surpassing 2 *z*-scores compared to their basal levels. However, the changes in plasma Aβ42/Aβ40, Aβ42, and Aβ40 were less pronounced (<1 *z*-score). As expected, CSF soluble Aβ drastically decreased before CSF p-tau became positive, and CSF t-tau levels parallelled those of CSF p-tau.

Throughout the whole Alzheimer’s continuum (Fig. 4D), we observed that plasma GFAP and p-tau181 continued to increase, while plasma NfL levels plateaued during the MCI+ and AD dementia phases. In contrast, the changes in plasma Aβ42/Aβ40, Aβ42, and Aβ40 were less prominent. Moreover, plasma biomarkers had smaller dynamic ranges than corresponding CSF biomarkers, except that the magnitude of CSF GFAP increase was not as high as that of plasma GFAP.

## Discussion

This is the first study to explore the dynamic changes of plasma biomarkers throughout the Alzheimer’s continuum in the Chinese Han population. The main findings are as follows: (1) there was a sharp and sustained increase in plasma GFAP as soon as CSF Aβ became positive, followed by CSF p-tau181 and t-tau, and, to a lesser extent, then plasma p-tau181. However, changes in plasma NfL, Aβ42/Aβ40, Aβ42, and Aβ40 were less pronounced; (2) plasma biomarkers exhibited smaller dynamic ranges than their CSF counterparts, except for GFAP which was the opposite; (3) plasma GFAP and p-tau181 outperformed other plasma biomarkers in tracking AD neuropathology and increased across age only in Aβ-positive individuals.

The cascade of biomarker changes in AD has been hypothesized to follow specific orderings from amyloid to tau, then to neurodegeneration [6], whereas this is not true for plasma biomarkers. Specifically, the earliest dynamic changes in plasma levels occurred in GFAP, which already increased early in the CU A+T− stage and continued to elevate as the disease progressed. This is consistent with previous studies that merely simplified the Alzheimer’s continuum into distinct stages [13, 17, 33], and we further reinforced the notion that plasma GFAP may be an early and sensitive biomarker of AD pathological changes by assessing the alterations of biomarkers as a function of CSF Aβ42/Aβ40 and p-tau181. Synthesizing our and former findings [13, 34], a model of biomarker trajectories in AD may therefore be updated (Fig. 4E). Among the plasma biomarkers studied, GFAP was the one that changed most prominently. However, the magnitude of its increases reported in a recent ALFA+ study was not as large as what we observed [22]. The heterogeneity in modeling approaches and biomarker cutoffs may help account for the discrepancy. Whether this discrepancy could be explained by ethnic heterogeneity remains to be verified by future large-scale studies. Supporting previous views [13, 17, 33, 35], plasma GFAP exerted a desirable accuracy in detecting the presence of Aβ burden and was strongly associated with AD pathologies, with no plasma biomarkers performing better than it. This further emphasizes the value of plasma GFAP in uncovering AD-related neuropathological changes. As a growing number of studies demonstrated the great promise of plasma p-tau231 and p-tau217 in the early identification of AD pathophysiology [12, 21–23], their inclusion in the analysis may yield intriguing results. However, limited by the current technical conditions, we are unable to quantify their concentrations. And this is one of the directions of our future research.

Plasma p-tau181 didn’t rise significantly until the latter CU A+T+ and symptomatic stages. It had a relatively steep increase and wide dynamic range throughout the Alzheimer’s continuum, closely following plasma GFAP. Decades of research have converged to demonstrate the specificity of plasma p-tau181 as an AD blood biomarker [9, 16, 36–40]. As a proof-of-principle, our plasma p-tau181 assay in Chinese people showed identical performances as previously described in other populations [10, 11, 36–39]. Specifically, it had similar but smaller dynamic ranges as CSF p-tau181, as well as close associations with AD pathologies and high accuracy in identifying amyloidosis. The consistent results obtained from diverse populations indirectly prove the reliability of our findings in Chinese cohorts. Plasma NfL barely increased during the preclinical AD stage, while increased markedly in later phases, closer to symptoms onset. This was in agreement with preceding observations [10, 41, 42], and with the fact that plasma NfL had modest accuracy in detecting advanced Aβ PET deposition while low accuracy in detecting earlier CSF Aβ pathology. Plasma Aβ42/Aβ40 decreased from the CU A+T+ stage, in a relatively flat trend. Its associations with AD pathologies were apparent in preclinical and prodromal stages, but not in advanced symptomatic stages. Given its slightly worse performance in discriminating Aβ status than previously reported [9], larger Chinese studies are needed to elucidate whether this difference is related to ethnic inheritance. In contrast, plasma Aβ42 and Aβ40 didn’t show pronounced changes throughout the entire Alzheimer’s continuum and lacked correlations with AD pathologies. This is in line with the previously reported dynamics across the clinical spectrum of AD [19] and the comparable concentrations of plasma Aβ in AD and controls [43], supporting that plasma Aβ levels might reflect peripheral Aβ generation more than they reflect AD brain pathology [19]. However, other studies demonstrated the potential of plasma Aβ markers in capturing early cerebral Aβ changes [10, 22, 23]. With the development of technology, recent studies have revealed that mass spectrometry-based methods outperform most immunoassays in the precise assays of plasma Aβ [44–46]. We thus hypothesize that the lack of significant changes in plasma Aβ levels may be an artifact attributable to the technique employed.

In the present study, we explored plasma biomarker changes in the context of established CSF biomarkers, which enabled us to make a direct comparison of their trajectories. Intriguingly, unlike plasma GFAP, CSF GFAP did not change noticeably across the AD continuum. The finding is surprising as neurologically related blood biomarkers are generally regarded as proxies of their CSF counterparts, but a recent study supports this idea [13]. The divergent clearance mechanisms in biofluids may be one possible interpretation [13]. In addition, plasma GFAP is very stable to freeze-thaw cycles [47], whereas CSF GFAP is far more sensitive over time [48], which may help explain the greater dynamic ranges of the former. In line with previous findings [10], other plasma biomarkers we tested exhibited smaller dynamic ranges than the corresponding CSF biomarkers, suggesting that the former are not sufficiently sensitive to reflect the pathological brain changes when compared to the latter. In this scenario, the early change of plasma GFAP before CSF p-tau181 and t-tau further underscores its potential as an ideal biomarker for tracking AD neuropathology. It is worth noting that although both NfL and t-tau are neurodegenerative biomarkers, plasma and CSF NfL had considerably smaller dynamic ranges than CSF t-tau. Therefore, they may provide different information concerning neurodegeneration. NfL may reflect age-related neuronal and axonal injury independent of Aβ pathology, while t-tau mainly reflects Aβ-dependent neurodegeneration [32, 42].

Plasma GFAP or p-tau181 increases as a function of age only occurred in Aβ-positive but not in negative individuals, suggesting that Aβ pathology underlies the glial or tau-related response. Of note, glial activation is not specific to AD pathophysiology, elevated blood GFAP levels could be observed in other neurological diseases (e.g., FTD, VaD), as our and other studies have shown [49–53]. The present study favors the finding that men had higher levels of plasma p-tau181 [54], whereas other plasma biomarkers were not found to differ by sex. Whether this indicates that men may have a greater susceptibility to tau deposition remains unclear. The effects of sex, often overlooked, do need further investigation to better understand AD pathogenesis and to design precision medicine strategies.

The early and prominent elevation of plasma GFAP, as observed in this study, has important implications for prevention trials of AD, which are increasingly seeking to enroll participants at initial neuropathological events. Substantial neurodegeneration and cognitive decline occur many years (even decades) after overt brain amyloidosis [55, 56], thus providing a window of opportunity for the initiation of disease-modifying therapies. Plasma p-tau181 has been proposed as a prescreening tool to detect Aβ pathology [11, 37, 38]. The present study shows that plasma GFAP may be superior at this task, like the current study [17], and has earlier and more marked changes than plasma p-tau181. This supports the idea of targeting glial markers early in the disease, whenever Aβ pathology emerges and before tau deposition. Moreover, plasma GFAP continued to increase along with disease progression, which implies that there may be late stages where treatment interventions can be effective. Such a biomarker could also be applied to track AD progression up to advanced disease stages. Presently, no effective treatments can cure AD or halt its progression. Elucidating the power of plasma GFAP as an estimator of target engagement is an urgent and arduous task for future clinical trials.

## Limitations

Strengths of our study include using well-characterized cohort data with multimodal biomarkers to determine the trajectories of plasma biomarkers from preclinical to symptomatic phases of AD and compare them directly with established CSF biomarkers. Besides, we tested the relations of plasma biomarkers with AD pathophysiology in different manners. Several caveats should be mentioned. First, this study was limited by its cross-sectional plasma data and could not provide direct insights into the longitudinal dynamics of plasma biomarker changes. Longitudinal studies are necessary to address this issue. Second, the sample size was relatively small. Large studies are needed to further validate and generalize our findings. Third, the hospital-based population selection in this study may include participants with more severe AD-related pathologies or comorbidities. Future studies of Asian populations in community or multicenter cohorts, as well as populations of other races or ethnicities, are warranted. Fourth, due to technical constraints, the definition of Aβ status in the present study is not uniform, that is, by CSF or PET assays. Future well-designed studies are warranted to circumvent this.

## Conclusions

In summary, we delineated the trajectories of plasma biomarkers in the context of established CSF biomarkers, in which plasma GFAP and p-tau181 indicated the most pronounced changes and were tightly linked to AD pathologies. As soon as CSF Aβ positivity has been attained, plasma GFAP began to continually increase across the entire Alzheimer’s continuum, followed by CSF p-tau181 and t-tau, and then plasma p-tau181. However, the changes in plasma NfL, Aβ42/Aβ40, Aβ42, and Aβ40 were less prominent. This study gave a new and comprehensive understanding of how plasma biomarkers changed and performed in the Chinese Han population, which has critical practical implications for future trials of AD prevention and treatment.

## Supplementary Information

**Additional file 1: Supplementary Methods. Table S1.** Demographic, clinical, and biomarker characteristics of non-AD dementia groups. **Figure S1.** Plasma biomarker group comparisons (including non-AD dementia). **Figure S2.** Correlations between plasma biomarkers. **Table S2.** Associations of plasma measures with Aβ PET. **Table S3.** Receiver operating curve analyses to discriminate Aβ status. **Figure S3.** Receiver operating curve analyses to discriminate diagnostic groups.

## Data Availability

The data used and analyzed in this study are available from the corresponding author upon reasonable request.

## Abbreviations

AD: Alzheimer’s disease
Αβ: Amyloid-β
p-tau: Phosphorylated tau
NfL: Neurofilament light
GFAP: Glial fibrillary acidic protein
CSF: Cerebrospinal fluid
PET: Positron emission tomography
CABLE: Chinese Alzheimer’s Biomarker and LifestylE
CU: Cognitively unimpaired
MCI: Mild cognitive impairment
FTD: Frontotemporal dementia
VaD: Vascular dementia
Simoa: Single-molecule array
t-tau: Total-tau
MRI: Magnetic resonance imaging
SD: Standard deviation
ROC: Receiver operating curve
AUC: Area under the curve
FDR: False discovery rate
