# Supplementary material for: The dynamics of plasma biomarkers across the Alzheimer’s continuum
Source: Alzheimers Res Ther. 2023 Feb 8;15:31. doi: 10.1186/s13195-023-01174-0 (PMC9906840; doi:10.1186/s13195-023-01174-0)
Supplement: Supplementary file 1 — Additional file 1: Supplementary Methods. Table S1. Demographic, clinical, and biomarker characteristics of non-AD dementia groups. Figure S1. Plasma biomarker group comparisons (including non-AD dementia). Figure S2. Correlations between plasma biomarkers. Table S2. Associations of plasma measures with Aβ PET. Table S3. Receiver operating curve analyses to discriminate Aβ status. Figure S3. Receiver operating curve analyses to discriminate diagnostic groups. [file 13195_2023_1174_MOESM1_ESM.docx]

**Supplementary Material**

Contents

Supplementary Methods…………………………………………………………………………………1

Table S1. Demographic, clinical, and biomarker characteristics of non-AD dementia groups……….…3

Figure S1. Plasma biomarker group comparisons (including non-AD dementia)....…………………4

Figure S2. Correlations between plasma biomarkers………………….………………………………....6

Table S2. Associations of plasma measures with Aβ PET…………………………………..………...7

Table S3. Receiver operating curve analyses to discriminate Aβ status………………….…………….14

Figure S3. Receiver operating curve analyses to discriminate diagnostic groups……………..…….…15

**Supplementary Methods**

*CSF collection, processing and storage*

Lumbar puncture was performed the same day of the blood drawing and, therefore, in fasting conditions. Following standard procedures, lumbar puncture was performed at the intervertebral space L3/L4, L4/L5 or L5/S1 using a standard needle. CSF specimens were centrifuged at 2000 × g for 10 minutes to eliminate cells and other insoluble materials, then aliquoted in volumes of 200 µL into enzyme free EP tubes and were snap frozen at −80 centigrade until subsequent processing. The thaw/freezing cycle was limited not to surpass two times. The duration between collection and freezing was less than 2 h. The time interval from sample collection to measurement was controlled within 6 months.

*CSF biomarkers measurements*

All measurements were performed by professional experimenters in Qingdao Municipal Hospital who were blind to clinical information. CSF Aβ42, Aβ40, p-tau181, and t-tau were determined with the ELISA kits (INNOTEST β-AMYLOID (1–42) [catalog number: 81583]; β-AMYLOID (1–40) [catalog number: 81585]; PHOSPHO-TAU (181p) [catalog number: 81581]; hTAU-Ag [catalog number: 81579]; Fujirebio, Ghent, Belgium). In addition to CSF samples, each plate also included blank control, run validation control, and internal control samples. If the quality control sample deviated, the plate would be reanalyzed. The standards and CSF samples were analyzed in duplicates, and the mean value of duplicate specimens was utilized for further statistical analyses. The within-batch coefficient of variation (CV) was < 5% (mean CV 4.5% for Aβ42, 3.7% for Aβ40, 2.5% for p-tau181, and 4.4% for t-tau). The inter-batch CV was < 20% (mean CV 5.3% for Aβ42, 3.4% for Aβ40, 2.4% for p-tau181, and 4.8% for t-tau). Quality control analysis showed that the levels of these CSF biomarkers were not related to sample collection time, storage time, intra-run and inter-run CV (all P values > 0.05).

*CSF amyloid-β and p-tau cutoffs derivation*

The optimal thresholds for CSF Aβ pathology positivity (A+) and tau pathology positivity (T+) were determined using Youden index. In an independent sample set, the derived cut-off points of 194.50 pg/mL for CSF Aβ42 and 57.50 pg/mL for p-tau181 best distinguished 18 CSF/PET-confirmed Aβ-positive AD dementia patients from 57 cognitively unimpaired subjects. Cases and control samples were matched (1-to-3 ratio) after adjustment for age, sex, years of education, and family history of dementia using the nearest-neighbor, propensity score-matching algorithm (caliper: 0.02 standard deviations). The thresholds showed excellent diagnostic accuracy (for CSF Aβ42: AUC = 0.936, 95% confidence interval = 0.881-0.991, sensitivity = 0.833, specificity = 0.912; for CSF p-tau181: AUC = 0.811, 95% confidence interval = 0.680-0.942, sensitivity = 0.667, specificity = 0.877). The cutoff point (0.063) for CSF Aβ42/Aβ40 was also calculated to model the relationship between biomarkers and the proxy of disease progression (CSF Aβ42/40). In addition, we quantified CSF Aβ42/Aβ40 levels by the Simoa assay and the cutoff point (0.046) was used to define Aβ status in ROC analysis.

**Table S1. Demographic, clinical, and biomarker characteristics of non-AD dementia groups**

| Characteristic | FTD N = 31 | VaD N = 37 |
| --- | --- | --- |
| Age, years | 59.61 (8.61) | 64.76 (8.18) |
| Male | 14 (45.16%) | 26 (70.27%) |
| Education, years | 8.59 (4.13) | 8.47 (3.96) |
| *APOE* ε4 carrier | 5 (23.81%) | 10 (32.26%) |
| Family history | 3 (11.11%) | 1 (2.78%) |
| MMSE score | 16.68 (7.98) | 16.39 (7.59) |
| MoCA score | 10.33 (7.05) | 10.53 (6.69) |
| **Plasma biomarkers** |  |  |
| GFAP, pg/ml | 131.88 (83.39) | 132.43 (55.21) |
| P-tau181, pg/ml | 2.04 (1.22) | 2.12 (1.12) |
| Aβ42/Aβ40 | 0.06 (0.02) | 0.06 (0.01) |
| Aβ42, pg/ml | 5.49 (1.77) | 5.41 (1.77) |
| Aβ40, pg/ml | 92.82 (34.79) | 95.13 (30.74) |
| NfL, pg/ml | 57.34 (37.12) | 73.76 (94.57) |

Continuous data are described as mean (standard deviations (SDs)), and categorical variables are presented as numbers (percentages).

Abbreviations: AD, Alzheimer’s disease; FTD, frontotemporal dementia; VaD, vascular dementia; *APOE*, *apolipoprotein E*; MMSE, Mini-Mental State Examination; MoCA, Montreal Cognitive Assessment; GFAP, glial fibrillary acidic protein; p-tau, phosphorylated tau; Αβ, amyloid-β; NfL, neurofilament light.

**Figure S1. Plasma biomarker group comparisons (including non-AD dementia)**


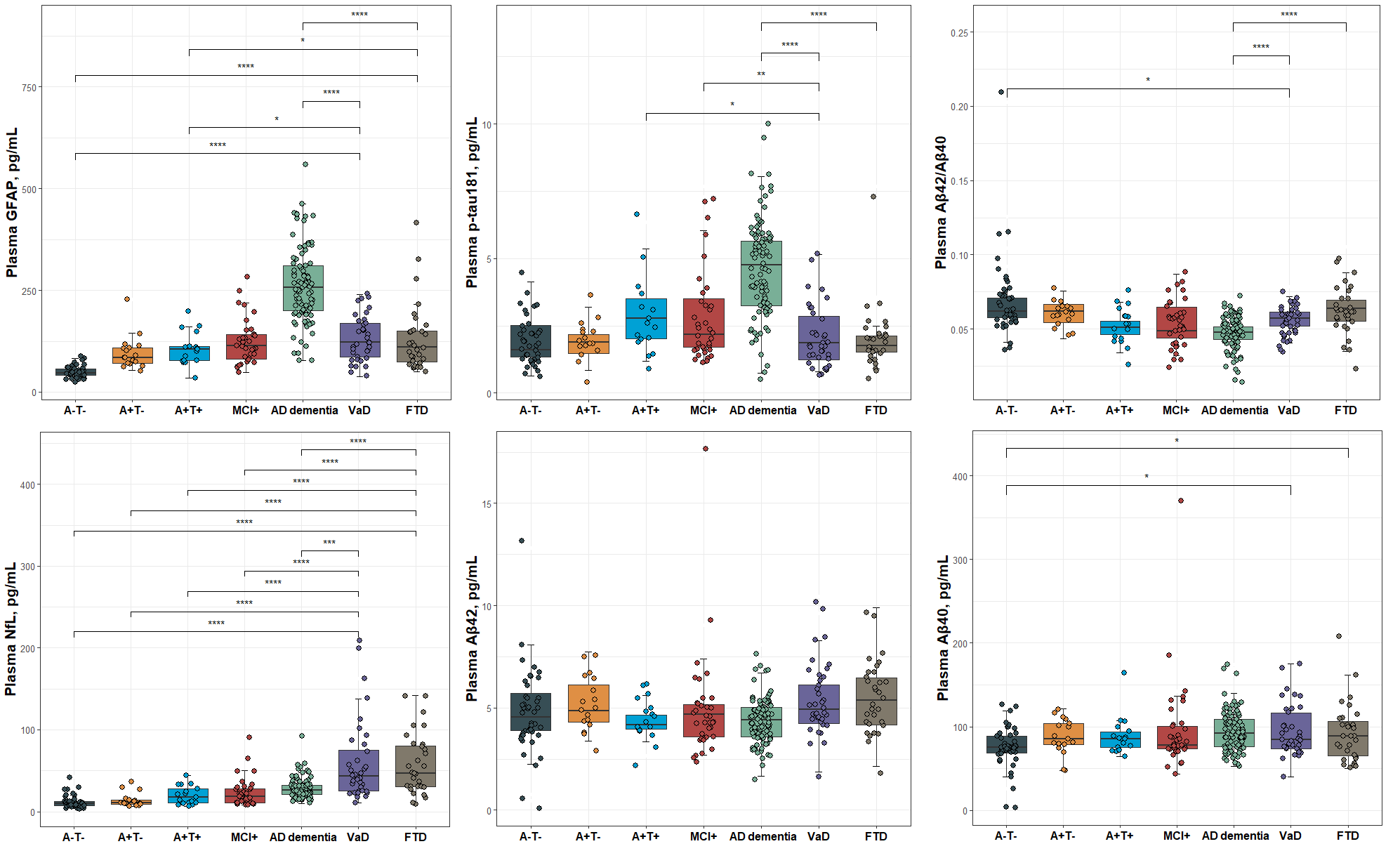


Note: Comparison results between VaD or FTD group versus other groups were displayed. Plasma levels of GFAP, p-tau181, Αβ42/Αβ40, NfL, Αβ42, and Αβ40 between groups were compared using one-way analysis of covariance controlling for age and sex, followed by FDR corrected pair-wise post hoc comparisons. Significance: ****p<0.0001, ***p<0.001, **p<0.01, *p<0.05, -: p≥0.05.

Abbreviations: AD, Alzheimer’s disease; GFAP, glial fibrillary acidic protein; MCI, mild cognitive impairment; VaD, vascular dementia; FTD, frontotemporal dementia; p-tau, phosphorylated tau; Αβ, amyloid-β; NfL, neurofilament light; FDR, false discovery rate.

**Figure S2. Correlations between plasma biomarkers**


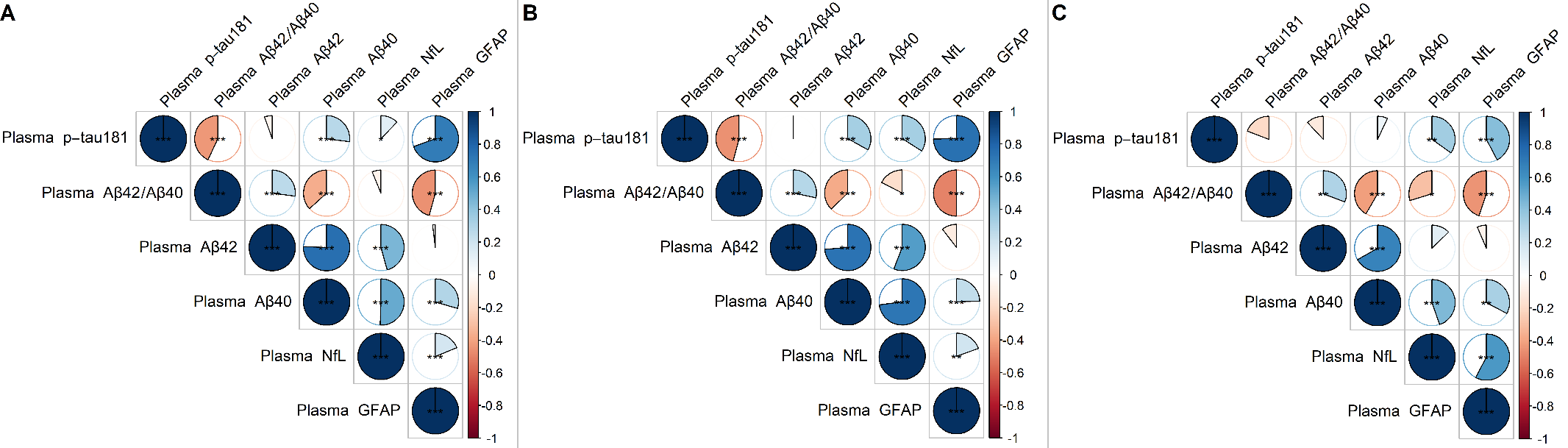


Note: The Spearman rank test was utilized for correlations between plasma biomarkers among participants within: (A) A–T–, Alzheimer’s continuum (A+T–, A+T+, MCI+, AD dementia), FTD, and VaD groups; (B) A–T– and Alzheimer’s continuum (A+T–, A+T+, MCI+, AD dementia) groups; (C) A–T– and preclinical AD (A+T–, A+T+) groups. In the heatmaps, different colors represent correlation coefficients. Significance: ***p<0.001, **p<0.01, *p<0.05, -: p≥0.05.

Abbreviations: p-tau, phosphorylated tau; Αβ, amyloid-β; NfL, neurofilament light; GFAP, glial fibrillary acidic protein; MCI, mild cognitive impairment; AD, Alzheimer’s disease; FTD, frontotemporal dementia; VaD, vascular dementia.

**Table S2. Associations of plasma measures with Aβ PET**

| Plasma measure | Brain region (Aβ PET) | β | Standard error | t | P | P value (FDR corrected) |
| --- | --- | --- | --- | --- | --- | --- |
| GFAP | Left accumbens | 0.137121431 | 0.108229574 | 1.266949751 | 0.209989221 | – |
| GFAP | Right accumbens | 0.476702202 | 0.139082703 | 3.427472947 | 0.001069768 | 0.029003905 |
| GFAP | Left amygdala | 0.041772814 | 0.04444551 | 0.93986578 | 0.350820264 | – |
| GFAP | Right amygdala | 0.005778465 | 0.040763493 | 0.141755897 | 0.887718109 | – |
| GFAP | Left caudate | 0.052026662 | 0.059817187 | 0.869761099 | 0.387681388 | – |
| GFAP | Right caudate | 0.072680009 | 0.056913663 | 1.277022152 | 0.206208182 | – |
| GFAP | Left hippocampus | -0.021403909 | 0.029764172 | -0.719116556 | 0.474686713 | – |
| GFAP | Right hippocampus | -0.028828967 | 0.02775885 | -1.038550451 | 0.302921721 | – |
| GFAP | Left pallidum | -0.033917927 | 0.038386765 | -0.883583883 | 0.380228048 | – |
| GFAP | Right pallidum | -0.038788014 | 0.041331279 | -0.93846633 | 0.351533126 | – |
| GFAP | Left putamen | 0.083623412 | 0.054165547 | 1.543848766 | 0.127555601 | – |
| GFAP | Right putamen | 0.079114491 | 0.053285146 | 1.484738185 | 0.142522171 | – |
| GFAP | Left thalamus | 0.009618157 | 0.033939661 | 0.28338988 | 0.777792679 | – |
| GFAP | Right thalamus | 0.007359198 | 0.032571034 | 0.225943034 | 0.821965739 | – |
| GFAP | Left bankssts | 0.19094675 | 0.066457543 | 2.873214116 | 0.005507146 | 0.029003905 |
| GFAP | Right bankssts | 0.175944195 | 0.06720411 | 2.618057076 | 0.011025419 | 0.037670182 |
| GFAP | Left caudal anterior cingulate | 0.112192694 | 0.06656796 | 1.685385788 | 0.096784547 | – |
| GFAP | Right caudal anterior cingulate | 0.165573079 | 0.075673242 | 2.188000338 | 0.032327588 | – |
| GFAP | Left caudal middle frontal | 0.161781968 | 0.068807532 | 2.35122469 | 0.021803037 | 0.045515112 |
| GFAP | Right caudal middle frontal | 0.160941082 | 0.067983564 | 2.367352813 | 0.020950442 | 0.045208849 |
| GFAP | Left cuneus | 0.195291278 | 0.068709117 | 2.8422906 | 0.006003198 | 0.029003905 |
| GFAP | Right cuneus | 0.167333772 | 0.068312528 | 2.449532733 | 0.017051415 | 0.041124001 |
| GFAP | Left entorhinal | -0.05742092 | 0.093479824 | -0.614260034 | 0.541221041 | – |
| GFAP | Right entorhinal | -0.066163046 | 0.090636274 | -0.729984174 | 0.468063456 | – |
| GFAP | Left frontal pole | 0.226484474 | 0.076100797 | 2.976111729 | 0.004116379 | 0.029003905 |
| GFAP | Right frontal pole | 0.238604582 | 0.084013342 | 2.840079643 | 0.006040197 | 0.029003905 |
| GFAP | Left fusiform | 0.129251465 | 0.053252188 | 2.42715785 | 0.018042691 | 0.042258793 |
| GFAP | Right fusiform | 0.103273701 | 0.052918404 | 1.951564927 | 0.055369084 | – |
| GFAP | Left inferior parietal | 0.180828168 | 0.064222703 | 2.815642414 | 0.006463381 | 0.029003905 |
| GFAP | Right inferior parietal | 0.163581167 | 0.065268494 | 2.506280683 | 0.014753076 | 0.039024266 |
| GFAP | Left inferior temporal | 0.189438965 | 0.062872108 | 3.013084367 | 0.003701996 | 0.029003905 |
| GFAP | Right inferior temporal | 0.135468232 | 0.0580039 | 2.335502122 | 0.022663802 | 0.045515112 |
| GFAP | Left insula | 0.09416788 | 0.052159808 | 1.805372441 | 0.075720111 | – |
| GFAP | Right insula | 0.074577972 | 0.054260176 | 1.374451335 | 0.174095448 | – |
| GFAP | Left isthmus cingulate | 0.062713485 | 0.060148305 | 1.042647585 | 0.301034119 | – |
| GFAP | Right isthmus cingulate | 0.089583014 | 0.059268255 | 1.511483902 | 0.135587456 | – |
| GFAP | Left lateral occipital | 0.207500636 | 0.064106118 | 3.236830462 | 0.001915937 | 0.029003905 |
| GFAP | Right lateral occipital | 0.169257588 | 0.067237747 | 2.517300106 | 0.01434061 | 0.039024266 |
| GFAP | Left lateral orbitofrontal | 0.118396147 | 0.060445723 | 1.958718359 | 0.054506215 | – |
| GFAP | Right lateral orbitofrontal | 0.106373808 | 0.059390439 | 1.791093149 | 0.078006983 | – |
| GFAP | Left lingual | 0.180004946 | 0.068036736 | 2.645702265 | 0.01024658 | 0.036531285 |
| GFAP | Right lingual | 0.161230621 | 0.070969557 | 2.271827926 | 0.026466664 | 0.049854837 |
| GFAP | Left medial orbitofrontal | 0.175090175 | 0.072468892 | 2.416073584 | 0.018552641 | 0.042258793 |
| GFAP | Right medial orbitofrontal | 0.209272725 | 0.072179746 | 2.899327541 | 0.005118036 | 0.029003905 |
| GFAP | Left middle temporal | 0.177957799 | 0.061816102 | 2.878825952 | 0.005421289 | 0.029003905 |
| GFAP | Right middle temporal | 0.150571648 | 0.059583297 | 2.527078128 | 0.013983359 | 0.039024266 |
| GFAP | Left paracentral | 0.139937736 | 0.061717433 | 2.267394004 | 0.026751376 | 0.049854837 |
| GFAP | Right paracentral | 0.150655136 | 0.059855178 | 2.516994213 | 0.014351918 | 0.039024266 |
| GFAP | Left parahippocampal | 0.140514731 | 0.067855537 | 2.070792412 | 0.042416468 | – |
| GFAP | Right parahippocampal | 0.100754886 | 0.057736724 | 1.745074515 | 0.085771777 | – |
| GFAP | Left pars opercularis | 0.142647646 | 0.064009035 | 2.22855486 | 0.029363238 | – |
| GFAP | Right pars opercularis | 0.123826876 | 0.066376794 | 1.865514557 | 0.066694066 | – |
| GFAP | Left pars orbitalis | 0.159171089 | 0.064717103 | 2.459490333 | 0.016626204 | 0.041124001 |
| GFAP | Right pars orbitalis | 0.141276845 | 0.060534545 | 2.333821863 | 0.022757556 | 0.045515112 |
| GFAP | Left pars triangularis | 0.156786233 | 0.065930636 | 2.378048248 | 0.020401564 | 0.045208849 |
| GFAP | Right pars triangularis | 0.158411987 | 0.063004796 | 2.514284581 | 0.014452436 | 0.039024266 |
| GFAP | Left pericalcarine | 0.274386656 | 0.088763393 | 3.091214133 | 0.00295079 | 0.029003905 |
| GFAP | Right pericalcarine | 0.2896502 | 0.090975914 | 3.183811943 | 0.00224523 | 0.029003905 |
| GFAP | Left postcentral | 0.123345589 | 0.05814904 | 2.121197325 | 0.037784227 | – |
| GFAP | Right postcentral | 0.084886974 | 0.057594602 | 1.473870318 | 0.145418455 | – |
| GFAP | Left posterior cingulate | 0.173988716 | 0.070585807 | 2.464924952 | 0.016398176 | 0.041124001 |
| GFAP | Right posterior cingulate | 0.244564134 | 0.083052988 | 2.944675916 | 0.004502173 | 0.029003905 |
| GFAP | Left precentral | 0.074727317 | 0.052152186 | 1.432870263 | 0.156761793 | – |
| GFAP | Right precentral | 0.070806785 | 0.05117486 | 1.383624396 | 0.171280576 | – |
| GFAP | Left precuneus | 0.207910319 | 0.069194907 | 3.004705527 | 0.003792353 | 0.029003905 |
| GFAP | Right precuneus | 0.227252417 | 0.071154594 | 3.19378418 | 0.002179498 | 0.029003905 |
| GFAP | Left rostral anterior cingulate | 0.15156646 | 0.065766757 | 2.304605951 | 0.024443767 | 0.047723545 |
| GFAP | Right rostral anterior cingulate | 0.16978921 | 0.067556902 | 2.513277017 | 0.014489976 | 0.039024266 |
| GFAP | Left rostral middle frontal | 0.21824796 | 0.072763702 | 2.999407023 | 0.003850545 | 0.029003905 |
| GFAP | Right rostral middle frontal | 0.198643183 | 0.070905768 | 2.8015095 | 0.006720417 | 0.029003905 |
| GFAP | Left superior frontal | 0.194036599 | 0.070900452 | 2.736747005 | 0.008022849 | 0.031327315 |
| GFAP | Right superior frontal | 0.198879154 | 0.069536606 | 2.860064149 | 0.005713272 | 0.029003905 |
| GFAP | Left superior parietal | 0.174092534 | 0.063321161 | 2.749357877 | 0.007752388 | 0.031327315 |
| GFAP | Right superior parietal | 0.172326757 | 0.06423797 | 2.682630817 | 0.009284389 | 0.03460545 |
| GFAP | Left superior temporal | 0.129411777 | 0.057646504 | 2.244919787 | 0.028236516 | – |
| GFAP | Right superior temporal | 0.107587533 | 0.058901281 | 1.826573741 | 0.072428156 | – |
| GFAP | Left supramarginal | 0.170214628 | 0.059718977 | 2.850260275 | 0.005871557 | 0.029003905 |
| GFAP | Right supramarginal | 0.154901768 | 0.060301695 | 2.568779659 | 0.012548325 | 0.039024266 |
| GFAP | Left temporal pole | 0.07100174 | 0.058490802 | 1.213895829 | 0.229248104 | – |
| GFAP | Right temporal pole | 0.038684462 | 0.074440431 | 0.519670041 | 0.605086344 | – |
| GFAP | Left transverse temporal | 0.089078019 | 0.070650318 | 1.260829695 | 0.2119473 | – |
| GFAP | Right transverse temporal | 0.083277142 | 0.069535133 | 1.197626844 | 0.235479129 | – |

| Plasma measure | Brain region (Aβ PET) | β | Standard error | t | P | P value (FDR corrected) |
| --- | --- | --- | --- | --- | --- | --- |
| P-tau181 | Left accumbens | 0.126103625 | 0.094325523 | 1.336898235 | 0.186220494 | – |
| P-tau181 | Right accumbens | 0.480619404 | 0.109619886 | 4.384418024 | 4.42E-05 | 0.0036244 |
| P-tau181 | Left amygdala | 0.015143433 | 0.03692227 | 0.410143608 | 0.683070358 | – |
| P-tau181 | Right amygdala | 0.004553459 | 0.033676846 | 0.135210387 | 0.892869892 | – |
| P-tau181 | Left caudate | 0.032023908 | 0.049547155 | 0.646331927 | 0.52037549 | – |
| P-tau181 | Right caudate | 0.059281938 | 0.047033782 | 1.260411872 | 0.212096937 | – |
| P-tau181 | Left hippocampus | -0.030886628 | 0.024384779 | -1.266635592 | 0.209876067 | – |
| P-tau181 | Right hippocampus | -0.013757821 | 0.023061109 | -0.596581076 | 0.552891607 | – |
| P-tau181 | Left pallidum | -0.03024185 | 0.031680965 | -0.954574777 | 0.34338449 | – |
| P-tau181 | Right pallidum | -0.029156458 | 0.034185855 | -0.85288076 | 0.396906076 | – |
| P-tau181 | Left putamen | 0.0616156 | 0.044918458 | 1.371721163 | 0.17494003 | – |
| P-tau181 | Right putamen | 0.064418491 | 0.044042685 | 1.462637696 | 0.148460219 | – |
| P-tau181 | Left thalamus | -0.00552296 | 0.028048018 | -0.196910886 | 0.844521271 | – |
| P-tau181 | Right thalamus | -0.005878795 | 0.026908945 | -0.218469897 | 0.827758165 | – |
| P-tau181 | Left bankssts | 0.154831405 | 0.055032873 | 2.81343492 | 0.006502923 | 0.04056004 |
| P-tau181 | Right bankssts | 0.158682889 | 0.054946854 | 2.887934035 | 0.005284572 | 0.04056004 |
| P-tau181 | Left caudal anterior cingulate | 0.086807147 | 0.055144206 | 1.574184364 | 0.120375713 | – |
| P-tau181 | Right caudal anterior cingulate | 0.092711735 | 0.063768247 | 1.453885583 | 0.150864563 | – |
| P-tau181 | Left caudal middle frontal | 0.132047966 | 0.056903323 | 2.320566868 | 0.023509321 | – |
| P-tau181 | Right caudal middle frontal | 0.121341523 | 0.056573464 | 2.144848743 | 0.035766862 | – |
| P-tau181 | Left cuneus | 0.099776458 | 0.05893426 | 1.69301283 | 0.095315829 | – |
| P-tau181 | Right cuneus | 0.093581017 | 0.057851204 | 1.61761572 | 0.110664029 | – |
| P-tau181 | Left entorhinal | 0.000730251 | 0.077454722 | 0.009428098 | 0.992506905 | – |
| P-tau181 | Right entorhinal | 0.014620939 | 0.075167154 | 0.194512338 | 0.846390865 | – |
| P-tau181 | Left frontal pole | 0.20986611 | 0.061737084 | 3.399352496 | 0.001167195 | 0.03190333 |
| P-tau181 | Right frontal pole | 0.193230825 | 0.06957753 | 2.777201536 | 0.007184601 | 0.04056004 |
| P-tau181 | Left fusiform | 0.113289727 | 0.043738637 | 2.590152228 | 0.011865804 | 0.04056004 |
| P-tau181 | Right fusiform | 0.0997407 | 0.043238455 | 2.306759083 | 0.024315817 | – |
| P-tau181 | Left inferior parietal | 0.151174655 | 0.052977904 | 2.853541644 | 0.005818134 | 0.04056004 |
| P-tau181 | Right inferior parietal | 0.134261285 | 0.053955269 | 2.488381346 | 0.015445913 | 0.043674651 |
| P-tau181 | Left inferior temporal | 0.155468111 | 0.051989736 | 2.990361599 | 0.003951813 | 0.04056004 |
| P-tau181 | Right inferior temporal | 0.109684509 | 0.047999911 | 2.285098158 | 0.025630501 | – |
| P-tau181 | Left insula | 0.09142938 | 0.04267099 | 2.142658982 | 0.035949607 | – |
| P-tau181 | Right insula | 0.086889488 | 0.044167463 | 1.967273687 | 0.053489345 | – |
| P-tau181 | Left isthmus cingulate | 0.081080136 | 0.049075591 | 1.652147933 | 0.103402769 | – |
| P-tau181 | Right isthmus cingulate | 0.092505811 | 0.048469957 | 1.908518511 | 0.060811109 | – |
| P-tau181 | Left lateral occipital | 0.118298565 | 0.055184499 | 2.143691946 | 0.035863301 | – |
| P-tau181 | Right lateral occipital | 0.10125029 | 0.056840899 | 1.781292899 | 0.079609604 | – |
| P-tau181 | Left lateral orbitofrontal | 0.124821998 | 0.04898684 | 2.548072059 | 0.013243347 | 0.041767479 |
| P-tau181 | Right lateral orbitofrontal | 0.110161389 | 0.048357113 | 2.278080336 | 0.026069738 | – |
| P-tau181 | Left lingual | 0.120731986 | 0.057246075 | 2.10900026 | 0.03886279 | – |
| P-tau181 | Right lingual | 0.1028905 | 0.05957666 | 1.727026982 | 0.088986688 | – |
| P-tau181 | Left medial orbitofrontal | 0.173460513 | 0.058661303 | 2.956983613 | 0.004347297 | 0.04056004 |
| P-tau181 | Right medial orbitofrontal | 0.205813058 | 0.057974032 | 3.550090499 | 0.000728087 | 0.029851567 |
| P-tau181 | Left middle temporal | 0.145433102 | 0.051139644 | 2.84384266 | 0.00597735 | 0.04056004 |
| P-tau181 | Right middle temporal | 0.12891785 | 0.04904193 | 2.628727076 | 0.01071865 | 0.04056004 |
| P-tau181 | Left paracentral | 0.097711216 | 0.051568925 | 1.894769293 | 0.06264224 | – |
| P-tau181 | Right paracentral | 0.090860143 | 0.050578983 | 1.796401135 | 0.077150259 | – |
| P-tau181 | Left parahippocampal | 0.103424511 | 0.056444176 | 1.832332728 | 0.071554948 | – |
| P-tau181 | Right parahippocampal | 0.122164735 | 0.046370545 | 2.634533102 | 0.010555005 | 0.04056004 |
| P-tau181 | Left pars opercularis | 0.11626171 | 0.052935252 | 2.196300279 | 0.031700376 | – |
| P-tau181 | Right pars opercularis | 0.105287093 | 0.054748092 | 1.923118952 | 0.05891651 | – |
| P-tau181 | Left pars orbitalis | 0.14314812 | 0.052995797 | 2.701122141 | 0.008834258 | 0.04056004 |
| P-tau181 | Right pars orbitalis | 0.121269199 | 0.049840415 | 2.43314986 | 0.017772283 | 0.047010555 |
| P-tau181 | Left pars triangularis | 0.142316148 | 0.053967011 | 2.637095256 | 0.010483516 | 0.04056004 |
| P-tau181 | Right pars triangularis | 0.128697844 | 0.052135387 | 2.468531456 | 0.01624841 | 0.044412321 |
| P-tau181 | Left pericalcarine | 0.196382956 | 0.074684184 | 2.629511974 | 0.010696393 | 0.04056004 |
| P-tau181 | Right pericalcarine | 0.198927484 | 0.076975569 | 2.584293778 | 0.012049458 | 0.04056004 |
| P-tau181 | Left postcentral | 0.081900544 | 0.048633427 | 1.684038079 | 0.097045987 | – |
| P-tau181 | Right postcentral | 0.043858054 | 0.048070387 | 0.91237155 | 0.364997238 | – |
| P-tau181 | Left posterior cingulate | 0.123057586 | 0.059048394 | 2.08401241 | 0.041156274 | – |
| P-tau181 | Right posterior cingulate | 0.145168114 | 0.070826317 | 2.049635215 | 0.044502786 | – |
| P-tau181 | Left precentral | 0.045206451 | 0.043404363 | 1.041518586 | 0.301553461 | – |
| P-tau181 | Right precentral | 0.037464385 | 0.042648941 | 0.878436462 | 0.382992979 | – |
| P-tau181 | Left precuneus | 0.184320137 | 0.056550325 | 3.259400165 | 0.001790018 | 0.036695369 |
| P-tau181 | Right precuneus | 0.171132823 | 0.05957062 | 2.872772249 | 0.005513959 | 0.04056004 |
| P-tau181 | Left rostral anterior cingulate | 0.145398619 | 0.053541445 | 2.715627466 | 0.008495222 | 0.04056004 |
| P-tau181 | Right rostral anterior cingulate | 0.132592617 | 0.056103951 | 2.363338319 | 0.021159837 | – |
| P-tau181 | Left rostral middle frontal | 0.173831513 | 0.06041017 | 2.877520666 | 0.005441148 | 0.04056004 |
| P-tau181 | Right rostral middle frontal | 0.156769716 | 0.05889129 | 2.662018737 | 0.009810761 | 0.04056004 |
| P-tau181 | Left superior frontal | 0.152914324 | 0.058881529 | 2.596982909 | 0.011654882 | 0.04056004 |
| P-tau181 | Right superior frontal | 0.146431155 | 0.058197294 | 2.516116198 | 0.01438442 | 0.042142588 |
| P-tau181 | Left superior parietal | 0.135556996 | 0.052656009 | 2.574387947 | 0.012365866 | 0.04056004 |
| P-tau181 | Right superior parietal | 0.115534067 | 0.054078696 | 2.136406295 | 0.036475912 | – |
| P-tau181 | Left superior temporal | 0.118716116 | 0.047185187 | 2.515961548 | 0.014390152 | 0.042142588 |
| P-tau181 | Right superior temporal | 0.104864047 | 0.048161037 | 2.177362729 | 0.033147349 | – |
| P-tau181 | Left supramarginal | 0.136960393 | 0.049496911 | 2.767049293 | 0.007387047 | 0.04056004 |
| P-tau181 | Right supramarginal | 0.116894877 | 0.050241178 | 2.326674668 | 0.023160215 | – |
| P-tau181 | Left temporal pole | 0.065298138 | 0.048188346 | 1.355060788 | 0.180161894 | – |
| P-tau181 | Right temporal pole | 0.007872872 | 0.061620005 | 0.127764869 | 0.898735665 | – |
| P-tau181 | Left transverse temporal | 0.061657825 | 0.058582713 | 1.052491808 | 0.296531599 | – |
| P-tau181 | Right transverse temporal | 0.049535039 | 0.057754963 | 0.857675887 | 0.39427197 | – |

Note: Plasma GFAP and p-tau181 showed remarkably positive associations with Aβ PET in MCI+ and AD dementia patients. “–” represents FDR-corrected p values>0.05.

Abbreviations: Αβ, amyloid-β; PET, positron emission tomography; FDR, false discovery rate; GFAP, glial fibrillary acidic protein; p-tau, phosphorylated tau; MCI, mild cognitive impairment; AD, Alzheimer’s disease.

**Table S3. Receiver operating curve analyses to discriminate Aβ status**

| CSF Aβ42+ vs – | Plasma GFAP | Plasma p-tau181 | Plasma Aβ42/Aβ40 | Plasma NfL | Plasma Aβ42 | Plasma Aβ40 |
| --- | --- | --- | --- | --- | --- | --- |
| AUC (95% CI) | 0.911 (0.866, 0.957) | 0.712 (0.63, 0.795) | 0.598 (0.507, 0.689) | 0.697 (0.616, 0.779) | 0.566 (0.476, 0.655) | 0.636 (0.551, 0.722) |
| Sensitivity | 0.86 | 0.52 | 0.43 | 0.54 | 0.50 | 0.71 |
| Specificity | 0.83 | 0.84 | 0.80 | 0.82 | 0.70 | 0.52 |

| Aβ PET + vs – | Plasma GFAP | Plasma p-tau181 | Plasma Aβ42/Aβ40 | Plasma NfL | Plasma Aβ42 | Plasma Aβ40 |
| --- | --- | --- | --- | --- | --- | --- |
| AUC (95% CI) | 0.971 (0.942, 1.000) | 0.916 (0.862, 0.97) | 0.543 (0.361, 0.724) | 0.802 (0.671, 0.933) | 0.523 (0.344, 0.701) | 0.671 (0.499, 0.842) |
| Sensitivity | 0.89 | 0.87 | 0.48 | 0.65 | 0.23 | 0.89 |
| Specificity | 1.00 | 1.00 | 0.69 | 0.85 | 0.93 | 0.46 |

| CSF Aβ42/Aβ40+ vs – | Plasma GFAP | Plasma p-tau181 | Plasma Aβ42/Aβ40 | Plasma NfL | Plasma Aβ42 | Plasma Aβ40 |
| --- | --- | --- | --- | --- | --- | --- |
| AUC (95% CI) | 0.966 (0.916, 1.016) | 0.966 (0.913, 1.019) | 0.519 (0.266, 0.772) | 0.814 (0.653, 0.976) | 0.508 (0.289, 0.726) | 0.708 (0.543, 0.874) |
| Sensitivity | 1.00 | 1.00 | 0.64 | 0.88 | 0.36 | 1.00 |
| Specificity | 0.85 | 0.85 | 0.63 | 0.82 | 0.88 | 0.48 |

Abbreviations: Αβ, amyloid-β; CSF, cerebrospinal fluid; GFAP, glial fibrillary acidic protein; p-tau, phosphorylated tau; NfL, neurofilament light; AUC, area under the curve; CI, confidence interval; PET, positron emission tomography.

**Figure S3. Receiver operating curve analyses to discriminate diagnostic groups**


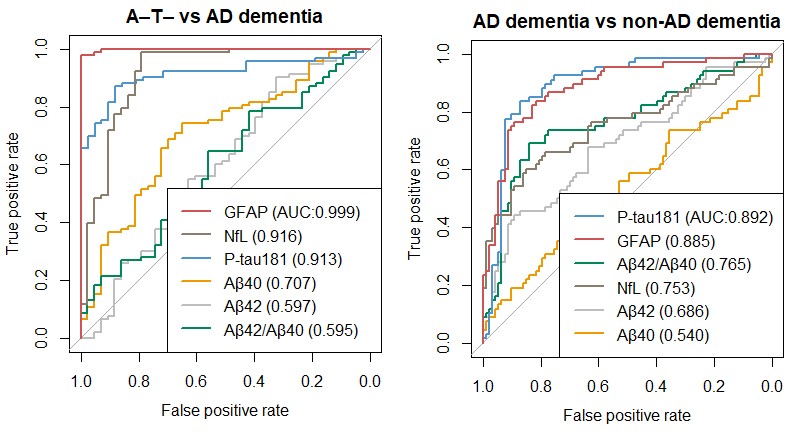


Abbreviations: AD, Alzheimer’s disease; GFAP, glial fibrillary acidic protein; AUC, area under the curve; NfL, neurofilament light; p-tau, phosphorylated tau; Αβ, amyloid-β.
